# Combination of vegetable soup and glucan demonstrates synergistic effects on macrophage-mediated immune responses

**DOI:** 10.1007/s10068-021-00888-x

**Published:** 2021-03-13

**Authors:** Ha-Nul Lee, Joo-Hee Choi, Ji-Yeon Park, Jae-Hun Ahn, Da Eun Jang, Jae Gun Shim, Jong-Hwan Park, Young-Min Kim

**Affiliations:** 1 Department of Integrative Food, Bioscience and Biotechnology, Chonnam National University, Gwangju, 61186 Republic of Korea; 2 Laboratory Animal Medicine, College of Veterinary Medicine, Chonnam National University, Gwangju, 61186 Republic of Korea; 3 Laboratory Animal Center, Daegu-Gyeongbuk Medical Innovation Foundation, Daegu, 41061 Republic of Korea; 4 Charmden Health Sciences Agricultural Co. 152, Nanosandan-ro, Jinwon-myeon, Jangseong-gun, Jeollanam-do Republic of Korea

**Keywords:** Vegetable soup, β-glucan, Synergistic effect, Mitogen-activated protein kinase, Immune response

## Abstract

Vegetable soup (VS), a plant-based functional food, has been used as a traditional folk medicine and is attracting attention for its ability to enhance the immune response. β-Glucan, a well-established and effective immunomodulator, has synergistic effects when used in combination with some bioactive compounds. In the present study, we aimed to evaluate the synergistic immunomodulatory effects of the combination of VS and β-glucan on macrophage-mediated immune responses. β-Glucan was demonstrated to synergistically enhance the VS-stimulated immune response, including the production of interleukin-6, tumor necrosis factor-α, and nitric oxide, mainly through the mitogen-activated protein kinase pathway in macrophages. In addition, this combination has the potential for further development in functional foods with immune-enhancing activity.

**Supplementary Information:**

The online version contains supplementary material available at 10.1007/s10068-021-00888-x.

## Introduction

The immune system is a host defense system that protects against diseases by regulating numerous biological structures and processes within an organism. Immune system disorders can lead to autoimmune diseases, inflammatory diseases, and cancer (O'Byrne and Dalgleish, 2001). One way to overcome defects in the immune system is by using nutraceutical ingredients to strengthen defensive reactions (Wichers, 2009). β-Glucan, a well-established and powerful immunomodulator, is naturally found in plants, yeast, fungi, and some bacterial species (Zeković et al., 2005). Recent studies have suggested that β-glucan has synergistic effects when combined with some bioactive molecules, such as vitamin C, humic acid, resveratrol, or coenzyme Q_10_ (Verlhac et al., 1996; Vetvicka et al., 2013; Vetvicka and Vetvickova, 2012; 2018).

Vegetable soup (VS) is a plant-based functional food consisting of radish roots and leaves, carrot roots, burdock roots, and shiitake mushrooms. It has been used as a traditional folk medicine and is attracting attention for its ability to improve the immune response of patients with cancer, lifestyle diseases, and autoimmune diseases. Various studies have suggested that VS has anti-inflammatory, anti-cancer, and immunomodulatory activities (Kim et al., 2018; Lim et al., 2017; Sim et al., 2010, 2015). Although many recent studies have focused on improving the immunological properties of VS, the scientific evidence for its biological effects and mechanisms underlying these effects remain unclear. Therefore, this study aimed to evaluate the synergistic immunomodulatory effects of the combination of VS and β-glucan on macrophage-mediated immune responses.

## Materials and methods

### Materials

VS was procured from Charmden Co., Ltd. (Gwangju, Korea). The product was manufactured by extracting five ingredients: radish roots (13 kg) and leaves (0.3 kg), carrot roots (5 kg), burdock roots (2.7 kg), and shiitake mushrooms (0.3 kg) were refluxed with water (100 L) at 110 °C for 30 min using a vacuum furnace. The extract was lyophilized to obtain a powder, and this power was dissolved in sterile distilled water for use in subsequent experiments. Curdlan, a linear β-1,3-glucan, was purchased from Wako Pure Chemicals (Osaka, Japan). Lipopolysaccharide (LPS) from *Escherichia coli* was purchased from InvivoGen (San Diego, CA, USA).

### Cell culture

Bone marrow-derived macrophages (BMDMs) were prepared as previously described (Celada et al., 1984). Briefly, BMDMs were cultured in complete Iscove’s modified Dulbecco’s medium (Gibco, Grand Island, NY, USA) supplemented with 30% L929 cell culture supernatant, 10% fetal bovine serum, 1% penicillin and streptomycin, 1% sodium pyruvate and 1% MEM non-essential amino acids in a 5% CO_2_ incubator (Panasonic Healthcare, Osaka, Japan) at 37 °C. After culture for 6 days, the adherent cells were collected and served as BMDMs.

### Measurement of cytokines

To determine the production of cytokines, the plated BMDMs were incubated with indicated concentrations of VS alone or combination with β-glucan for 24 h. The concentrations of interleukin (IL)-6 and tumor necrosis factor (TNF)-α in the culture supernatants were determined by using commercial enzyme-linked immunosorbent assay (ELISA) kits following the manufacturer’s instructions (R&D Systems, Minneapolis, MN, USA).

### Nitric oxide (NO) assay

Nitrite accumulation in the culture supernatants was measured by the Griess reaction (Kim et al., 1995). The BMDMs were incubated with indicated concentrations of VS alone or combination with β-glucan supplemented with 100 ng/mL interferon (IFN)-γ for 24 h. An aliquot of the culture supernatant was mixed with an equal volume of Griess reagent containing with 1% sulfanilamide and 0.1% naphthylethylenediamine dihydrochloride in 5% phosphoric acid, and incubated at room temperature for 10 min. The absorbance was measured, and the concentration of nitrite was calculated with reference to a standard curve obtained with sodium nitrite.

### Immunoblotting

The plated BMDMs were incubated with 1 mg/mL VS alone or combination with 3 mg/ml β-glucan for indicated times. After remove the culture medium, the cells were lysed in a lysis buffer containing 1% Nonidet-P40, protease inhibitor cocktail (Roche, Mannheim, Germany), and phosphatase inhibitor cocktail (**Sigma-Aldrich**, St. Louis, MO, USA). Denature protein samples were separated by 10% sodium dodecyl sulfate polyacrylamide gel electrophoresis and transferred to polyvinylidene difluoride membranes. The membranes were immunoblotted with the primary antibodies specific to the target proteins, and the proteins detected using an enhanced chemiluminescence reagent (BioRad, Hercules, CA, USA).

### Statistical analysis

The data obtained from three independently repeated experiments were presented as mean ± standard deviation. Statistical analysis was performed using one-way analysis of variance followed by Tukey's post-hoc analysis (GraphPad Prism 5; GraphPad Software Inc., La Jolla, CA, USA), or followed by Duncan post-hoc analysis (R statistical software). Differences were considered significant when *p* values were less than 0.05.

## Results and discussion

**Fig. 1:**
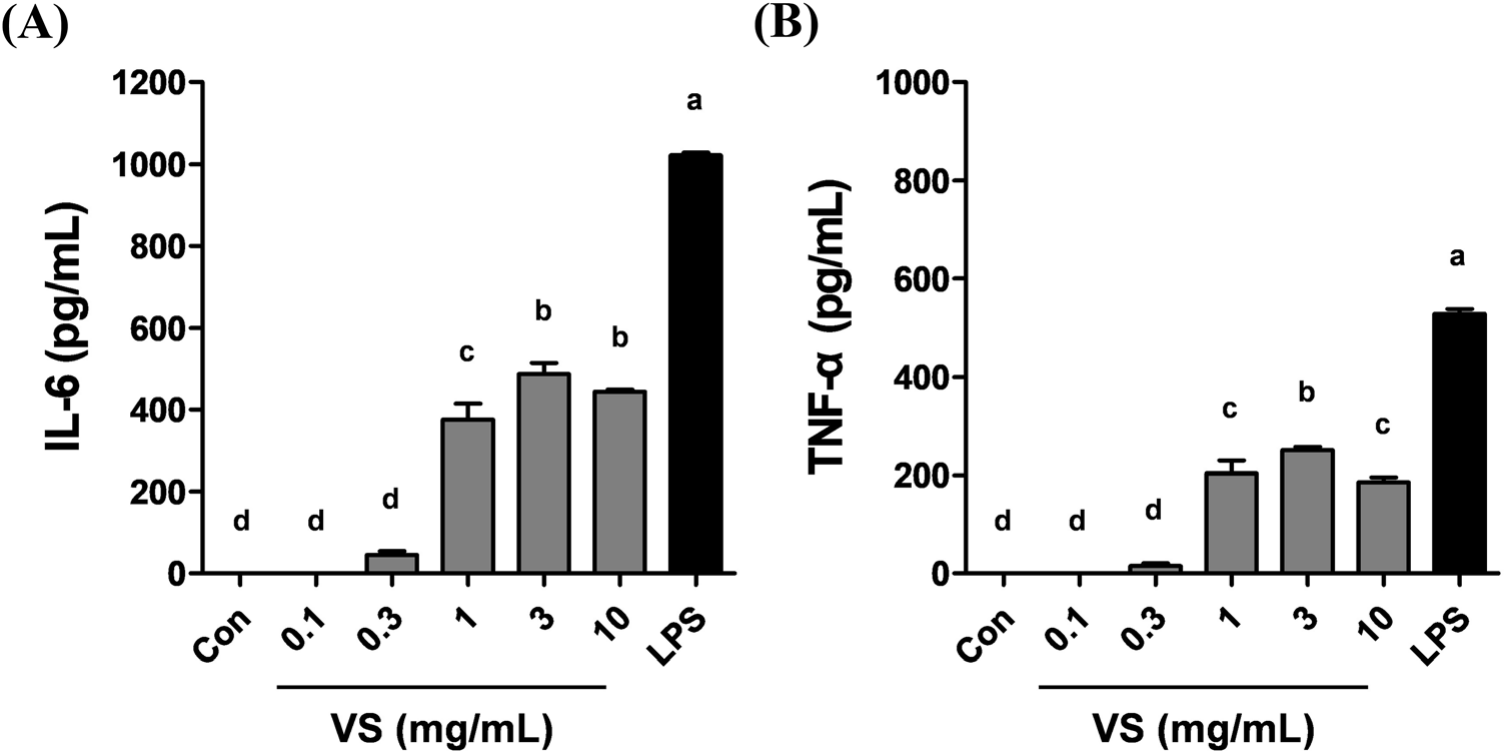
Effect of VS on cytokine production in macrophages. BMDMs were treated with various doses of VS in the range of 0.1–10 mg/mL for 24 h. The levels of IL-6 (**A**) and TNF-α (**B**) in the culture supernatants were determined by ELISA. LPS (100 ng/mL) was used as the positive control. All values are represented as the mean ± standard deviation of triplicate experiments. Different letters indicate statistically significant difference (*p* < 0.05)

Macrophages play an important role in effective innate and adaptive immune responses (Rooijen and Annemarie Sanders, 1997). Cytokines, such as IL-6 and TNF-α, are produced in activated macrophages and critically regulate the immune response (Cheng et al., 2008). The measurement of cytokine production is one method for evaluating the enhancement of innate immunity (Yu et al., 2013). Firstly, we investigated whether VS could stimulate macrophage activity and consequently regulate cytokine production. BMDMs were treated with various doses of VS in the range of 0.1–10 mg/mL for 24 h, and the cytokines in the culture supernatants were quantified by ELISA. LPS, an outer membrane component of Gram-negative bacteria, was used as a positive control because it is known to activate a number of cellular signals in macrophages (Guha and Mackman, 2001). We found that the cytokine production in BMDMs incubated with VS was significantly enhanced (Fig. 1). The levels of IL-6 and TNF-α increased progressively with the increase in the concentration of VS (ranging from 0.1 to 3 mg/mL). However, above 3 mg/mL, cytokine production no longer increased; rather, a slight decrease was observed at 10 mg/mL VS. This indicated that there was a limit to the stimulation of macrophage activity by VS and that additive substances should be identified to enhance the VS-stimulated immune response in macrophages.

VS is a complex extract consisting of five ingredients: radish roots and leaves, carrot roots, burdock roots, and shiitake mushrooms. It was wondered which ingredients in VS could enhance the immune response of macrophages. BMDMs were incubated with 1 mg/mL individual ingredients of VS for 24 h, and the cytokines in the culture supernatants were quantified by ELISA. While the cytokine production was considerably enhanced by VS, the ingredients had little effect on macrophages (Fig. S1). In recent studies, the oligo- or poly-saccharides isolated from burdock roots, carrot roots, and shiitake mushrooms have shown the immunomodulatory activities in immune cells (Morales et al., 2020; Sun et al., 2020; Zhang et al., 2019). It was suggested that the compounds of VS induced the immune response and that their combination synergistically improved the response of macrophages.

**Fig. 2:**
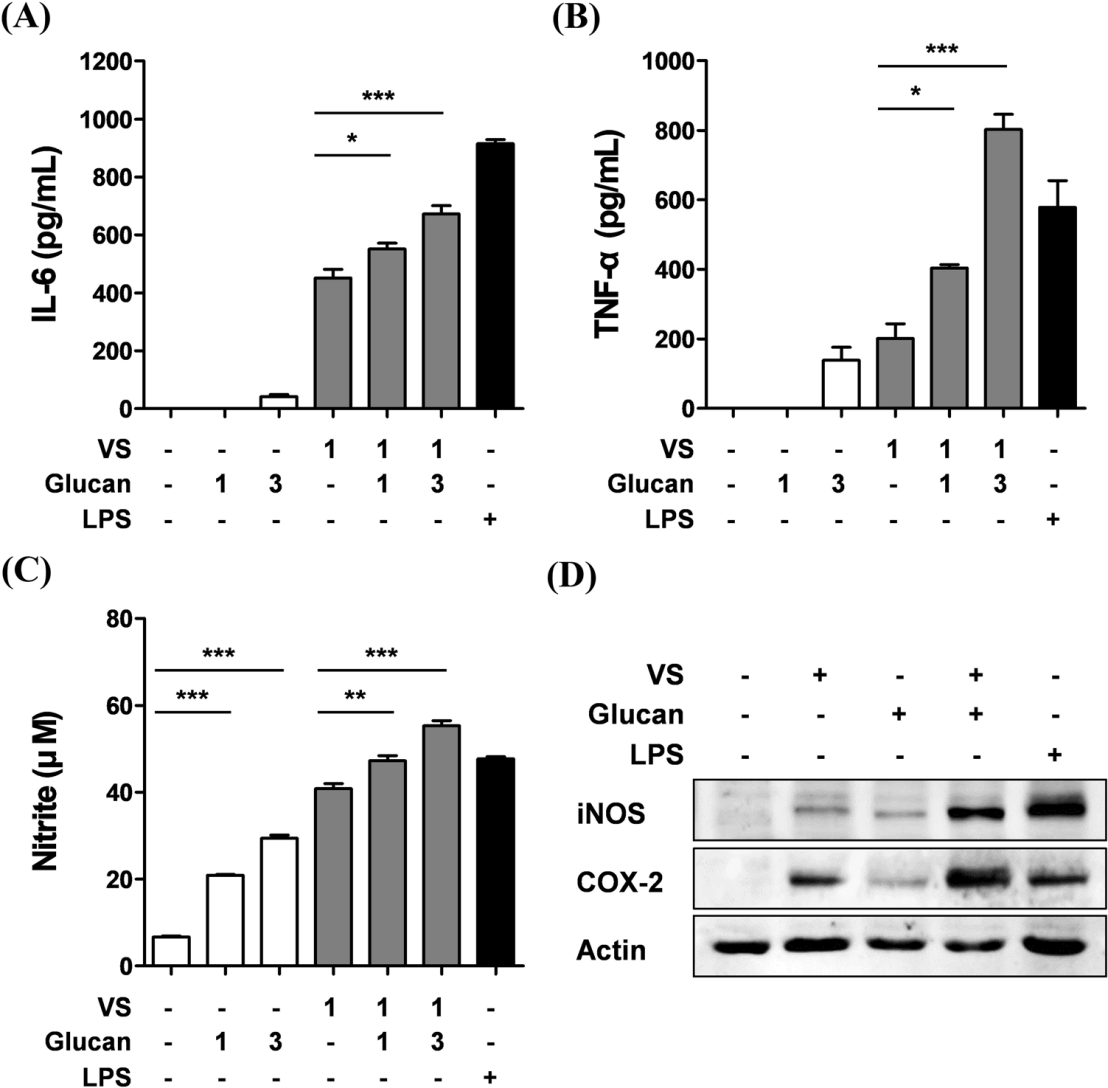
Effect of β-glucan on the VS-stimulated immune response in macrophages. (**A**, **B**) The release of cytokines in BMDMs treated with a combination of 1 or 3 mg/mL VS and 1 or 3 mg/mL β-glucan was determined by ELISA. (**A**) IL-6, (**B**) TNF-α. (**C**) The nitrite contents in IFN-γ-stimulated BMDMs treated with a combination of 1 or 3 mg/mL VS and β-glucan were determined by the Griess assay. LPS (100 ng/mL) was used as the positive control. All values are represented as the mean ± standard deviation of triplicate experiments. *p < 0.05, **p < 0.01, ***p < 0.001. (**D**) Activation of the iNOS and COX-2 pathways in BMDMs treated with 1 mg/mL VS and/or 3 mg/mL β-glucan was determined by immunoblotting. Actin was used as the loading control

Various previous studies have demonstrated that β-glucan is an effective immunomodulator and has significant synergetic effects with numerous bioactive molecules (Verlhac et al., 1996; Vetvicka et al., 2013; Vetvicka and Vetvickova, 2012; 2018). In addition, the combination of *Grifola frondosa*-derived β-glucan and *Withania somnifera* extract has potent pleiotropic biological effects for immune health and stress reduction (Vetvicka and Vetvickova, 2011). Therefore, we investigated the synergetic effect of VS and β-glucan on macrophage-mediated immune responses. BMDMs were incubated with a combination of VS and β-glucan for 24 h. Subsequently, the cytokines in the cell supernatants were quantified by ELISA. Surprisingly, β-glucan markedly and synergistically enhanced VS-stimulated cytokine production; however, β-glucan alone had little effect on the induction of IL-6 and TNF-α release in macrophages (Fig. 2A, B). In particular, in BMDMs treated with 1 mg/mL VS and 3 mg/mL β-glucan, the level of TNF-α increased by four-fold compared to that in case of BMDMs treated with VS only. These results demonstrated that β-glucan improved and synergistically enhanced VS-stimulated cytokine production in macrophages. Furthermore, macrophages can exert effects on adaptive immunity through cytokine signaling (Arango Duque and Descoteaux, 2014). TNF-α plays a central role in the innate response and can indirectly stimulate adaptive immunity by inducing macrophages to release cytokines such as IL-6, which are directly related to both innate and adaptive immunity (McInnes and Schett, 2011). IL-6 promotes the differentiation of B cells into plasma cells and activates cytotoxic T cells (Okada et al., 1988; Tanaka et al., 2014). It means that the synergistically enhanced cytokine production of the macrophages might lead to activate the adaptive immune cell.

As a major signal transduction mediator of macrophages, NO plays an important role in the immune system (Chen et al., 2010; Lee and Jeon, 2005). The effect of VS and its combination with β-glucan on NO production in IFN-γ-stimulated macrophages was determined by the Griess assay. The nitrite concentration in the culture supernatants was increased after treatment with VS or β-glucan for 24 h (Fig. 2C). Moreover, β-glucan significantly enhanced VS-stimulated NO production in macrophages. In particular, BMDMs treated with 1 mg/mL VS and 3 mg/mL β-glucan exhibited a greater release of NO than the LPS-treated positive control. As shown in Fig. 2D, using immunoblotting, we also confirmed that VS- or β-glucan-stimulated NO production was attributed to an increase in the activation of inducible NO synthase (iNOS), and this consequently increased the induction of cyclooxygenase (COX)-2. Moreover, this combination strongly activated the iNOS and inducible COX-2 pathways. These results showed that VS stimulated NO production and NO-related pathways, and this effect was dramatically enhanced by combined treatment with β-glucan in macrophages. Collectively, these results suggested that VS and β-glucan have a significant synergistic effect on macrophage-mediated immune responses. The previous study demonstrated that a short-term oral application of the combination of maitake (β-glucan rich) and shiitake mushrooms (α-glucan rich) strongly stimulated the immune responses (Vetvicka and Vetvickova, 2014). Therefore, the synergistic effect is speculated as a result of β-glucan enhancing the immune responses stimulated by the bioactive compounds such as polysaccharides with immunomodulatory activities contained in VS.

**Fig. 3:**
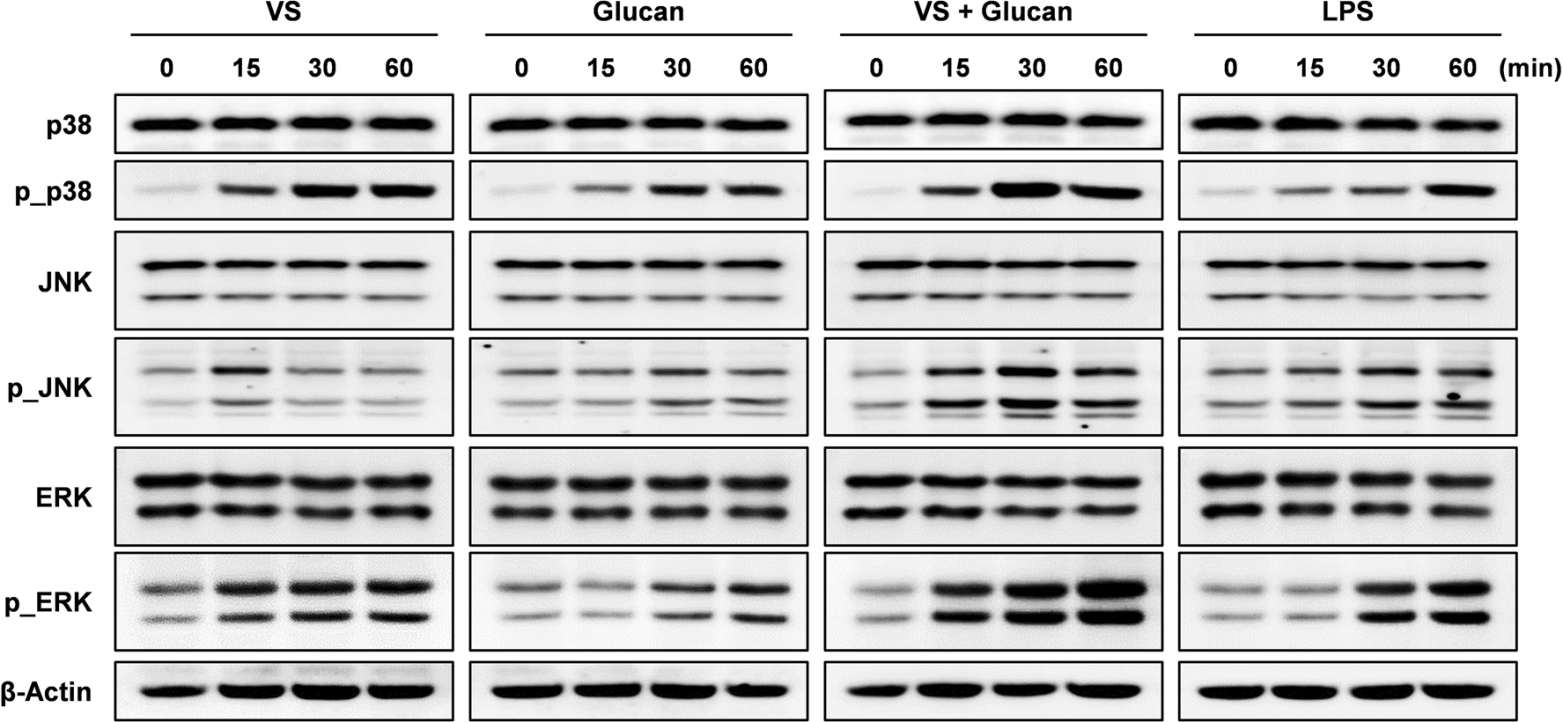
Effect of the combination of VS and β-glucan on the activation of MAPK signaling in macrophages. BMDMs were treated with 1 mg/mL VS and/or 3 mg/mL β-glucan for the indicated times. The components of MAPK (p38, JNK, and ERK) pathways were determined by immunoblotting. LPS (100 ng/mL) was used as the positive control. Actin was used as the loading control

The mitogen-activated protein kinase (MAPK) and nuclear factor (NF)-κB pathways play an important role as the mediators of cellular responses to extracellular signals (Baeuerle and Baltimore, 1996; Surh et al., 2001). Accordingly, we investigated whether the synergistic effect of VS and β-glucan could affect the activation of several important components of MAPK (p38, JNK, and ERK) and NF-κB (IκB and p65) signaling. BMDMs were treated with 1 mg/mL VS and/or 3 mg/mL β-glucan for the indicated time periods, and the aforementioned signaling pathway components was determined by immunoblotting. The activation of the components of MAPK and NF-kB pathways were slightly and slowly enhanced in β-glucan-treated BMDMs (Figs. 3 and S2). In comparison, VS stimulated the activation of these pathways faster and to a greater extent. Surprisingly, β-glucan significantly enhanced VS-stimulated MAPK activation, and this activation was stronger than that induced by LPS treatment (Fig. 3). While NF-kB activation was ineffective compared to that in the control, the phosphorylated activation of p38, JNK, and ERK was synergistically improved by co-treatment with VS and β-glucan in BMDMs. In particular, β-glucan achieved prolonged and more potent JNK activation, which was only stimulated slightly and temporarily by VS treatment. Moreover, the activation of other MAPK components, p38 and ERK, were significantly enhanced by the combined treatment. The MAPK pathway is an important signaling pathway in immune responses and is involved in transcriptional regulation of cytokines, iNOS, and COX-2 expression in macrophages (Arbabi and Maier, 2002; Uto et al., 2005). Particularly, JNK plays an important role in activating AP-1, which is an important regulator of gene expression (Johnson and Lapadat, 2002). Overall, these results demonstrated that β-glucan synergistically enhanced VS-stimulated immune response, including cytokine and NO production, mainly through the MAPK pathway in macrophages.

In conclusion, the present study suggests that β-glucan synergistically improves the immunomodulatory properties of VS in macrophages, and this combination has potential for further development in functional foods with immune-enhancing activity. VS is a complex comprising numerous bioactive compounds, one or more of which may have synergistic immunological effects when combined with β-glucan. While some of these compounds may have been reported previously, others may be new. Thus, further studies are required to investigate the exact bioactive components derived from VS and to determine the molecular mechanism underlying macrophage-mediated immune responses.

## Supplementary Information

Below is the link to the electronic supplementary material.

**Fig. S1.** Effect of VS and its ingredients on cytokine production in macrophages. BMDMs were treated with 1 mg/mL individual ingredients of VS for 24 h. The levels of IL-6 (A) and TNF-α (B) in the culture supernatants were determined by ELISA. LPS (100 ng/mL) was used as the positive control. All values are represented as the mean ± standard deviation of triplicate experiments. Different letters indicate statistically significant difference (*p* < 0.05)

Supplementary file1 (JPG 419 KB)

**Fig. S2.** Effect of the combination of VS and β-glucan on the activation of NF-κB signaling in macrophages. BMDMs were treated with 1 mg/mL VS and/or 3 mg/mL β-glucan for the indicated times. The activation of the components of NF-κB pathways was determined by immunoblotting. LPS (100 ng/mL) was used as the positive control. Actin was used as the loading control

Supplementary file2 (JPG 740 KB)
