# Supplementary figures and images for: Combination of vegetable soup and glucan demonstrates synergistic effects on macrophage-mediated immune responses
Source: Food Sci Biotechnol. 2021 Mar 13;30(4):583–8. doi: 10.1007/s10068-021-00888-x (PMC8050188; doi:10.1007/s10068-021-00888-x)

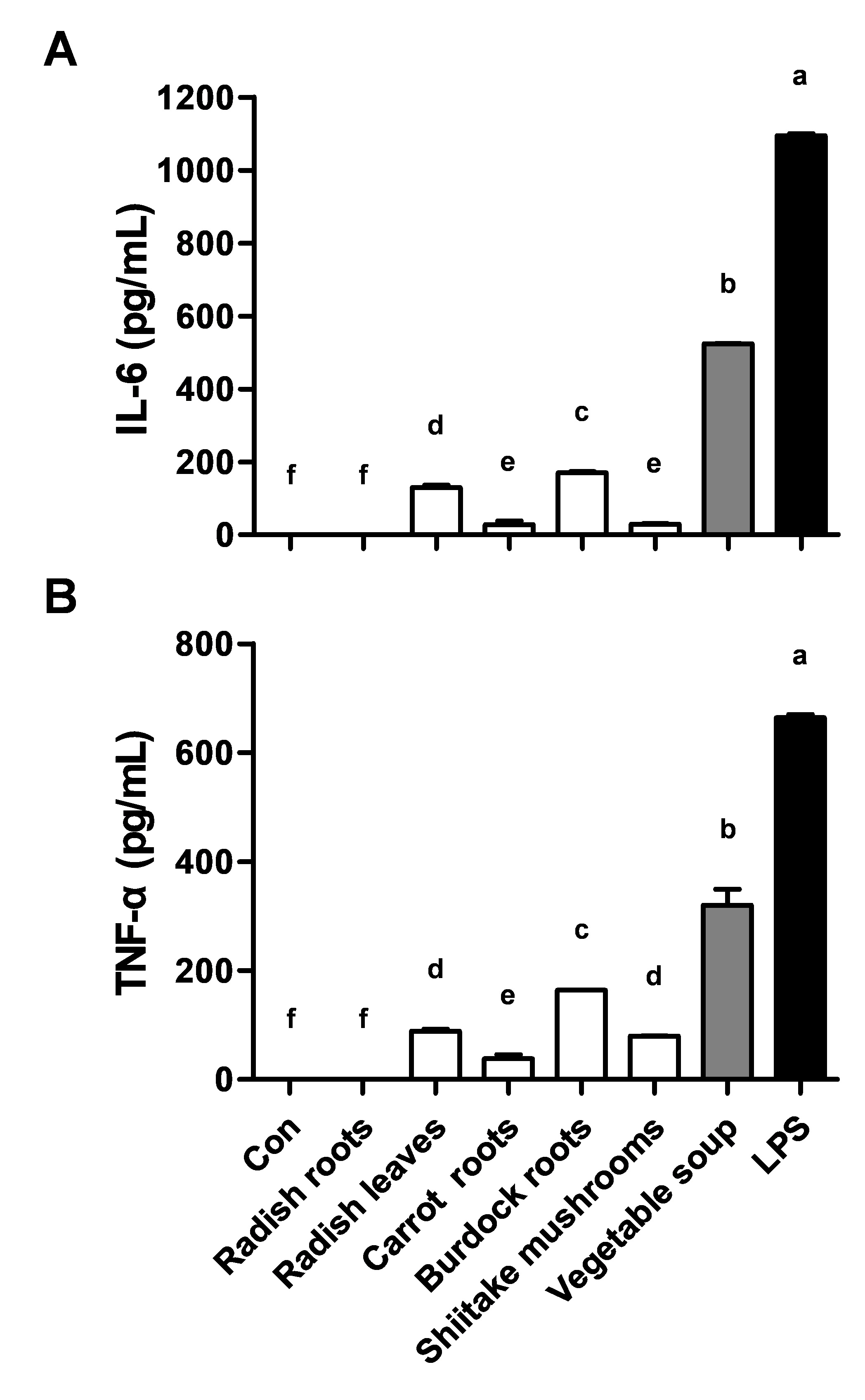

Supplement: Supplementary file 1 — Supplementary file1 (JPG 419 KB) [file 10068_2021_888_MOESM1_ESM.jpg]

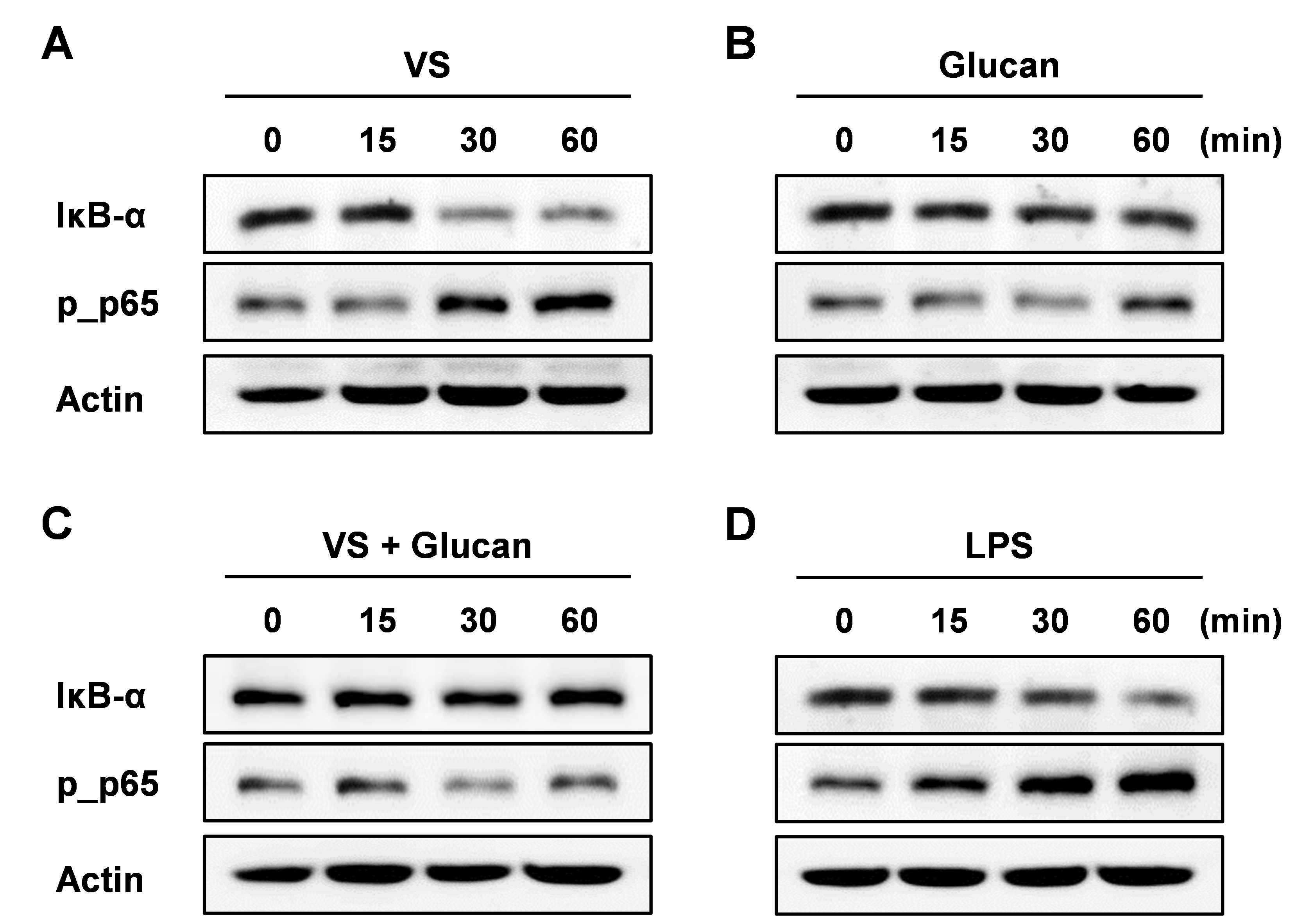

Supplement: Supplementary file 2 — Supplementary file2 (JPG 740 KB) [file 10068_2021_888_MOESM2_ESM.jpg]
